# Comparative analysis of the terpenoid biosynthesis pathway in *Azadirachta indica* and *Melia azedarach* by RNA-seq

**DOI:** 10.1186/s40064-016-2460-6

**Published:** 2016-06-21

**Authors:** Yuwei Wang, Xiang Chen, Jin Wang, Hang Xun, Jia Sun, Feng Tang

**Affiliations:** Key Laboratory of Bamboo and Rattan Science and Technology of State Forestry Administration, International Center for Bamboo and Rattan, No. 8, Futong East Street, Wangjing Area, Chaoyang District, Beijing, 100102 People’s Republic of China

**Keywords:** *Azadirachta indica*, *Melia azedarach*, Azadirachtin, Terpenoid biosynthesis, RNA-seq

## Abstract

*Azadirachta indica* (neem) is the only source of azadirachtin, which is known for its insecticide activity. *Melia azedarach* is a related species of *A. indica*, widely distributed in the south of China. In this study, the leaf transcriptomes of these two *Meliaceae* plants were sequenced. More than 40 million clean reads were generated from each library. About 80 % of *A. indica* reads were mapped to the neem genome, while 93 % of *M. azedarach* reads were mapped to its assembled transcripts and unigenes dateset. After mapping and assembly, 225,972 transcripts and 91,607 unigenes of *M. azedarach* were obtained and 1179 new genes of *A. indica* were detected. Comparative analysis of the annotated differentially expressed genes (DEG) showed that all six DEGs involved in terpenoid backbone biosynthesis were up-regulated in *A. indica*. Chemical analysis of the two plants revealed *A. indica* leaves contained 2.45 % total terpenoid and nearly 20–50 µg azadirachtin per gram, whereas azadirachtin was not detected in *M. azedarach* and total terpenoid content was reached 1.67 %. These results give us a better insight into the transcriptomes differences between *A. indica* and *M. azedarach*, and help us to understand the terpenoid biosynthesis pathway in vivo.

**Electronic supplementary material:**

The online version of this article (doi:10.1186/s40064-016-2460-6) contains supplementary material, which is available to authorized users.

## Background

*Azadirachta indica* A. Juss (neem tree) and *Melia azedarach* Linn. are two species in the *Meliaceae* family, that have a close relationship in phylogenetic systematics. However, in chemical analysis of different tissues in the two species, azadirachtin (a kind of triterpene) was found in nearly all parts of *A. indica*, whereas no azadirachtin or its derivatives were found in *M. azedarach* (Tan and Luo 2011). Azadirachtin is the most important activated compound in the neem tree, having effective biological functions and huge commercial value (Atawodi and Atawodi 2009). Azadirachtin is an efficient environmentally friendly plant-derived pesticide, that interfere with insect growth and development (Qiao et al. 2014). A study on the biology and mortality of rice leaffolder larvae treated with neem extract showed azadirachtin was a potent pesticide and caused almost 100 % larval mortality at a 1 ppm concentration (Senthil Nathan et al. 2006). Because of its broad spectrum toxicity to insects, azadirachtin has been registered as a pesticide in many countries. NeemAzal, a kind of azadirachtin-based commercial insecticide, was shown to have a strong inhibitory effect on *Rhyzopertha dominica*, *Sitophilus oryzae* and *Tribolium confusum* (Athanassiou et al. 2005). Besides insecticidal activity, neem tree extracts also have many pharmaceutical functions, such as anticancer, antimicrobial, anti-inflammatory and antidiabetic activities (Thoh et al. 2010; Soares et al. 2014). However, despite plentiful information on the usefulness of the neem tree, there have been few molecular studies on this plant, especially about the biosynthesis of azadirachtin in vivo. Fortunately, the whole *A. indica* genome and five transcriptomes (including stem, leaf, flower, root and fruit) have been sequenced (Krishnan et al. 2011, 2012), which has established a solid foundation for molecular biological research on the neem tree. Though the mechanism of azadirachtin biosynthesis is unknown, a lot of research has focused on the synthesis of azadirachtin, including chemosynthesis (Veitch et al. 2007), hairy root culture (Srivastava and Srivastava 2013), callus culture (Rodrigues et al. 2014) and cell line culture in vitro (Singh and Chaturvedi 2013).

Using the omics strategy to study the metabolic pathways which active phytomedicinals are produced has become a hotspot of secondary metabolite research in recent years (Misra 2014), and lies at the intersection of chemistry, biology, mathematics and computer science. Using this method, researchers sequenced the root transcripts of American ginseng and found that one CYP450 and four UDP-glycosyltransferases were most likely involved in ginsenoside biosynthesis (Sun et al. 2010). Based on their different terpenoid products, in this research we comparatively analyzed the leaf transcriptomes of *A. indica* and *M. azedarach* by specific RNA-seq, and screen for genes related to azadirachtin biosynthesis. This study will help us to understand the formation of azadirachtin in vivo, and also provides potential targets for regulation with existing research strategies to harvest greater amounts of environment-friendly biopesticides.

## Methods

### Plant materials

Seeds of *A. indica* were obtained from Yuanmou Desert Ecosystem Research Station, Yuanmou County, Yunnan Province, China, while seeds of *M. azedarach* were collected from Dong’an Forest Park, Chaohu County, Anhui Province, China. The two *Meliaceae* plants were identifed by Yanping Zhang (Research Institute of Resources Insects of the Chinese Academy of Forestry, China) and Yiming Hu (Anhui Academy of Forestry, China), respectively. Sampling of plant materials did not affect the local ecology and was performed with permission from local administrative departments. After seed germination, the plants were grown in a greenhouse at 28 °C with a 16 L:8 D photoperiod. Leaves of *A. indica* and *M. azedarach* were collected from the plants and immediately frozen in liquid nitrogen and stored at −80 °C until analysis.

### Azadirachtin and total terpenoid quantitative analysis in leaves

One gram of cryopreserved leaf powder was weighted into a 5-mL centrifuge tube, 3 mL methanol was added for extraction, and the tube was mixed using a homogenizer (Fluko, Essen, Germany) for 2 min. The tube was then centrifuged at 4000 rpm for 3 min and the supernatant was removed to a 10-mL volumetric flask. The extraction was repeated three times, and the supernatants were combined and dried using a Termovap Sample Concentrator (Organomation, Berlin, MA, USA). The concentrated sample was purified with an ENVI-Carb SPE (Supelco, Bellefonte, PA, USA) according to the manufacturer’s instructions and then concentrated to 1 mL. Last, a Waters model 2695 high performance liquid chromatography (HPLC) apparatus equipped with a model 2996 photodiode array detector (PAD) (Waters, Milford, MA, USA), was used to detect the azadirachtin content in each sample; the detection parameters were as follows: X Terra^®^ RP18 column (4.6 × 250 mm, 5 µm), 10 μL sample size, mobile phase of methanol–water (6:4), 1 mL/min velocity, 210–360 nm detection wavelength. Total terpenoid content assay was performed with Ghorai’s methods (Ghorai et al. 2012).

### RNA extraction and strand-specific library construction

Total RNAs were isolated using an EASYspin Plus Complex Plant RNA Kit (Aidlab, Beijing, China), and then unwanted cytoplasmic, mitochondrial, and chloroplast ribosomal RNAs were removed using Ribo-Zero™ rRNA Removal Kits (Illumina, San Diego, CA, USA). The quality of the collected RNAs was initially estimated with a Nanodrop 8000 UV–Vis Spectrophotometer (Thermo Fisher Scientific, Waltham, MA, USA) and then the integrity of the RNA samples was precision detected with an Agilent 2100 Bioanalyzer (Agilent, Santa Clara, CA, USA) according to the user’s guide. Strand-specific cDNA libraries of the two *Meliaceae* plants were constructed for transcriptome sequencing using the NEBNext^®^ Ultra™ Directional RNA Library Prep Kit for Illumina^®^ (NEB, Ipswich, MA, USA) according to the directions. First, purified mRNAs were randomly broken into shorter fragments, and random primers were added by hybridization. Next, first-strand cDNA was synthesized using the fragments as templates, and the second-strand cDNA was synthesized using dUTP instead of dTTP for labelling. The fragments were then purified, end-repaired, dA-tailed and ligated with adapters. Finally, the second-strand was selectively removed using the USER enzyme (NEB) while the first-strand was left for the PCR amplification.

### Sequencing and quality control of the data

Based on sequencing by synthesis technologies, the test qualified libraries were then sequenced using an Illumina Hiseq™ 2500 with 125 bp pair-end reads at the Biomarker Technologies Company in Beijing, China. Via base calling, huge numbers of raw reads were acquired. The quality scores of the bases (Q-values), reflecting the probability of mismatched bases, and the base distribution was determined to evaluate sequencing quality. Finally, reads with adaptors, reads with unknown nucleotides larger than 10 % and low quality reads in which the percentage of bases with Q-values <10 was more than 50 % were removed, leaving only the clean reads. The datasets for each sample were deposited in the Short Read Archive (SRA) database of the National Institutes of Health (NIH).

### Reads assembly, mapping and new gene detection

The *A. indica* reference genome (364M) and gene set were downloaded from the NCBI FTP site (ftp://ftp.ncbi.nlm.nih.gov/genomes/all/GCA_000439995.3_AzaInd2.1), and also could be browsed online from the official website of the Ganit Labs, Bio-IT Centre, Institute of Bioinformatics and Applied Biotechnology, (http://115.119.161.46:96/cgi-bin/gb2/gbrowse/neemV2/) using Gbrowse 2.0. After the neem genome was prepared, clean reads from the *A. indica* libraries (Y1–3) were aligned to the reference genome using TopHat2 (Kim et al. 2013). Briefly, the mapping process was divided into two steps. First, the reads were aligned against the neem genome, and then split into smaller segments, which were aligned to the genome. After that, the Cufflinks software was used to assemble the mapped reads (Trapnell et al. 2010), which were compared with the original genome annotation information to screen for new genes.

For the *M. azedarach* libraries (K1–3), because there was little genome information for *M. azedarach*. it was necessary to assemble the clean reads before annotation. Based on the known related species: *Citrus Clementina* (301M), *Citrus sinensis* (328M) and *A. indica* (364M), the genome size of *M. azedarach* was likely between 300 to 400M. The assembler program Trinity (Grabherr et al. 2011), which is better than other de novo transcriptome assembly programs in many respects, was used for this process. Briefly, Trinity first extends the clean reads set in *k*-mer space and breaks ties. Next, it overlaps linear sequences by overlaps of *k*-1 to build graph components. Last, it builds a De Bruijn graph and compacts it to get the transcripts and unigenes. After reads assembly was finished, the clean reads were mapped to transcripts and unigenes sets using Bowtie2 (Langmead et al. 2009).

### Functional annotation of new genes and unigenes

Before further bioinformatics analysis, it was necessary to test the quality of the transcriptome libraries. Normally, detecting the distribution of inserted segments in the genes or unigenes, the length profile of the inserted fragments and the saturation curve map, which are common methods to assess the quality of libraries. For functional annotation, the new genes and unigenes were aligned to five public databases: Gene Ontology (GO), Kyoto Encyclopedia of Genes and Genomes (KEGG), Eukaryotic Orthologous Groups (KOG), Swiss-Prot and Non-redundant Protein (Nr) databases.

### Quantitative analysis of gene expression

After using Bowtie to align the clean reads and unigenes set, RNA-seq by expectation maximization (RSEM) was used to accurately quantify the expression levels of transcripts from the RNA-seq data (Li and Dewey 2011). Fragments per kilobase of transcript per million fragments mapped (FPKM) values were used to reflect the expression levels of transcripts or genes.

In this study, to compare the differentially expressed genes (DEG) in the two *Meliaceae* plants, we needed to screen for orthologous genes was needed. OrthoMCL was used to analyze the homologous proteins among the known protein sequences in *A. indica* and predicted protein sequences in *M. azedarach* (Li et al. 2003). After that, according to methods established by Brawand et al. (2011), a corresponding degree of scaling was used to normalize the gene expression levels in the different species (Brawand et al. 2011). The fold changes of gene expression were assessed by the log_2_ ratio (FPKM-Y/FPKM-K).

## Results and discussion

### Sequence analysis and assembly

To obtain a comprehensive overview of the differences in terpenoid biosynthesis between the two *Meliaceae* plants, three cDNA libraries from *A. indica* and three from *M. azedarach* were constructed. For convenient analysis of the RNA-seq data, the three *A. indica* libraries were named Y1–Y3 and the *M. azedarach* libraries were named K1–K3. The six libraries were sequenced using the Illumina Hiseq™ 2500 sequencing platform. After removing the adapter and low quality reads, 46,203,176, 43,956,038 and 56,227,214 clean reads were acquired from Y1 to Y3, respectively (Table 1), while 51,341,436, 45,384,504 and 41,737,868 clean reads were obtained from K1 to K3, respectively (Table 2). The number of reads from each library was ten-fold higher than in the previous sequencing (Krishnan et al. 2012).

**Table 1:** Alignment statistics of three *A. indica* samples Y1–Y3

Sample	Y1	Y2	Y3
Raw reads	47,035,150	44,757,094	57,245,842
Clean reads	46,203,176	43,956,038	56,227,214
Clean bases	5,820,550,242	5,537,418,082	7,083,668,168
GC content (%)	43.22	43.19	43.19
≥Q30 (%)	91.89	91.77	91.44

Map to scaffold	Reads number	Percentage	Reads number	Percentage	Reads number	Percentage
Mapped reads	37,542,577	81.26	35,920,248	81.72	46,256,906	82.27
Unique mapped reads	23,258,114	50.34	22,121,124	50.33	28,636,295	50.93
Multiple mapped reads	14,284,463	30.92	13,799,124	31.39	17,620,611	31.34

Y1–3 stands for the three *A. indica* libraries

**Table 2:** Alignment statistics of three *M. azedarach* samples K1–K3

Sample	K1	K2	K3
Clean reads	51,341,436	45,384,504	41,737,868
Clean bases	6,467,847,204	5,717,571,930	5,257,985,424
GC content (%)	42.98	43.13	43.41
≥Q30 (%)	91.60	91.27	91.56

Mappped to transcript and unigene	Reads number	Percentage	Reads number	Percentage	Reads number	Percentage
Mapped reads	47,752,662	93.01	42,408,364	93.44	38,963,326	93.35

K1–3 stands for the three *M. azedarach* libraries

Though *A. indica* and *M. azedarach* are related species, there were great differences between the two *Meliaceae* plants at the transcriptome level. Initially, we planned to align the reads of three *M. azedarach* libraries to the neem genome database, but the low mapping ratio (<20 %) made it necessary to perform de novo assembly. Using the assembler Trinity (Grabherr et al. 2011), 225,972 transcripts and 91,607 unigenes were acquired, with corresponding N50 lengths of 2628 and 1321 bp, respectively (Table 3).

**Table 3:** Assembly statistics of *Melia azedarach*

Length range	Transcript	Unigene
200–300	38,222 (16.91 %)	33,321 (36.37 %)
300–500	29,928 (13.24 %)	22,795 (24.88 %)
500–1000	32,359 (14.32 %)	17,392 (18.99 %)
1000–2000	48,480 (21.45 %)	10,234 (11.17 %)
2000+	76,983 (34.07 %)	7865 (8.59 %)
Total number	225,972	91,607
Total length	365,526,044	67,998,977
N50 length	2628	1321
mean length	1617.57	742.29

### Mapping of reads to the *A. indica* genome dataset, and *M. azedarach* transcripts and unigenes

To identify the corresponding genes of the sequences in each library, the clean reads were mapped to the *A. indica* genome. The mapping results showed that more than 80 % of reads from each library were matched to the reference genome while about 50 % were uniquely matched. Based on the reference genome, the cufflinks software was used to splice the mapped reads of the *A. indica* libraries (Y) and 53,381 genes were acquired. After the mapped reads were assembled, 1179 new genes were screened out by comparison with the original neem genome annotation information.

In order to test the quality of the assembly, the clean reads were mapped to the unigenes and transcripts dataset. The results are shown in Table 2; more than 93 % of clean reads were mapped. Next, the mapped reads were used to detect the saturation of genes in each library; the saturation curve is shown in Additional file 1: Figure S1.

### Analysis of differential genes expression in the leaves of the two *Meliaceae* plants

**Fig. 1:**
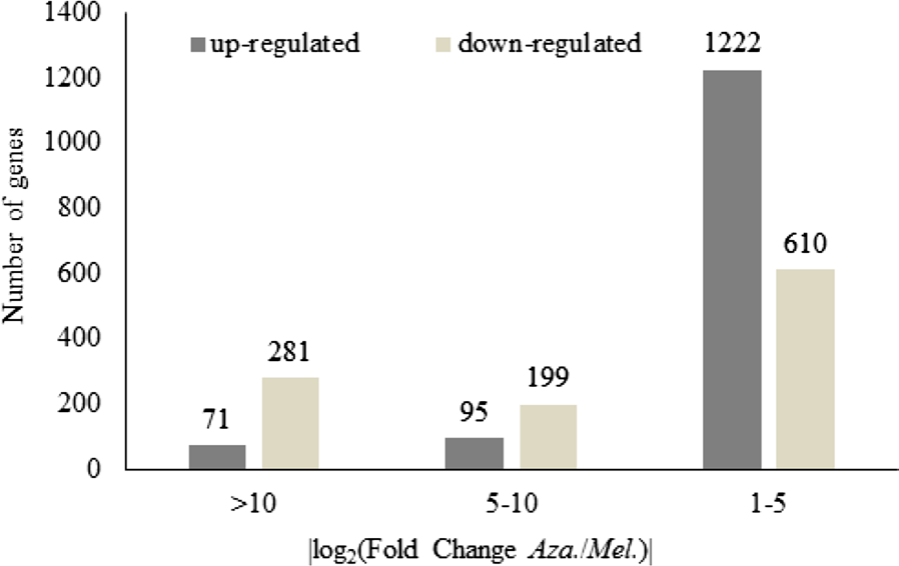
The distribution of fold-changes in differentially expressed gene numbers

For comparative analysis of the DEGs in the libraries of the two speices, protein homology analysis was performed. Using the Orthomcl software (version 2.0.9), 3867 orthologous genes were identified in the two species (Additional file 2: Table S1). Comparison of the expression of orthologous genes showed that the majority of genes were expressed at different levels in the two species. In total, 2478 genes showed more than two-fold expression changes (log_2_(Fold Change)| ≥ 1); of these, 1388 genes were up-regulated and 1090 were down-regulated (Additional file 3: Table S2). Notably, among the 2478 DEGs, 352 genes showed more than 2^10^-fold changes in expression level, including 71 up-regulated and 281 down-regulated genes. The distribution of fold-changes in DEG numbers between the *A. indica* (Y) and *M. azedarach* (K) libraries is shown in Fig. 1.

### Functional annotation of all genes (*A. indica*), unigenes (*M. azedarach*) and DEGs

For the further study, all genes (53,381) of *A. indica* including new genes (1179), the unigenes (91,607) of *M. azedarach* and the 2478 DEGs were aligned with the GO, KEGG, KOG, Swiss-Prot and Nr databases. The number of annotated genes in each database is listed in Table 4, and detailed annotation information for the DEGs, new genes and unigenes is shown in Additional file 3: Table S2, Additional file 4: Table S3, Additional file 5: Table S4.

**Table 4:** The annotation statistics of all genes, new genes, unigenes and DEGs

	Total	Annotated	GO	KEGG	KOG	Swiss-Prot	Nr
All genes (*A. indica*)	53,381	53,159	29,854	–	–	36,657	53,154
New genes (*A. indica*)	1179	1055	672	517	–	648	1054
Unigenes (*M. azedarach*)	91,607	53,732	33,191	21,238	30,597	33,198	53,216
DEGs (*A. indica* vs *M. azedarach*)	2478	2459	1346	763	1200	1611	2431

– Stands for not alignment with this database

From the GO annotation, 16,901 (*A. indica*), 15,649 (*M. azedarach*) and 1346 (DEGs) annotated genes were categorized into three main groups. For cellular components, genes associated with cell parts and organelles were the most highly represented, while genes related to catalytic activity and binding represented the largest proportion of genes with molecular functions. For biological processes, the most represented GO term was metabolic process, followed by cellular process and single-organism process. More information on the functional categorization of genes in *A. indica*, the unigenes in *M. azedarach* and the DEGs is shown in Additional file 6: Figure S2.

**Fig. 2:**
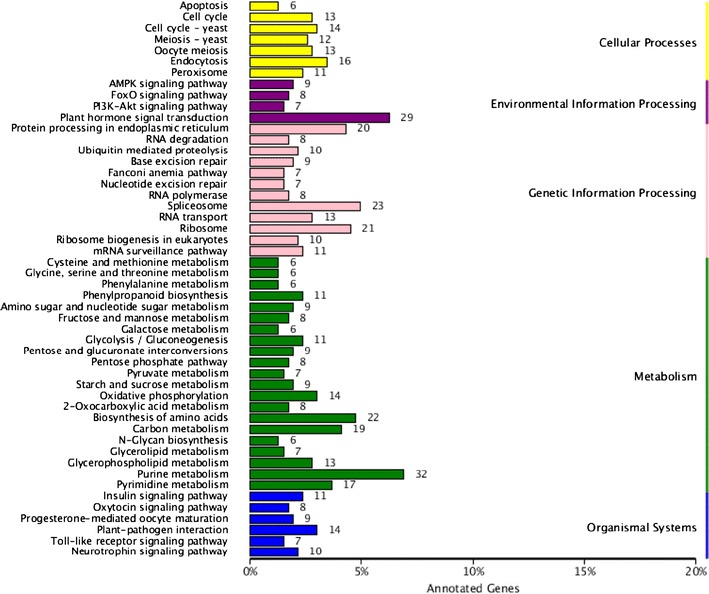
The type classification of DEGs with KEGG annotation results

**Fig. 3:**
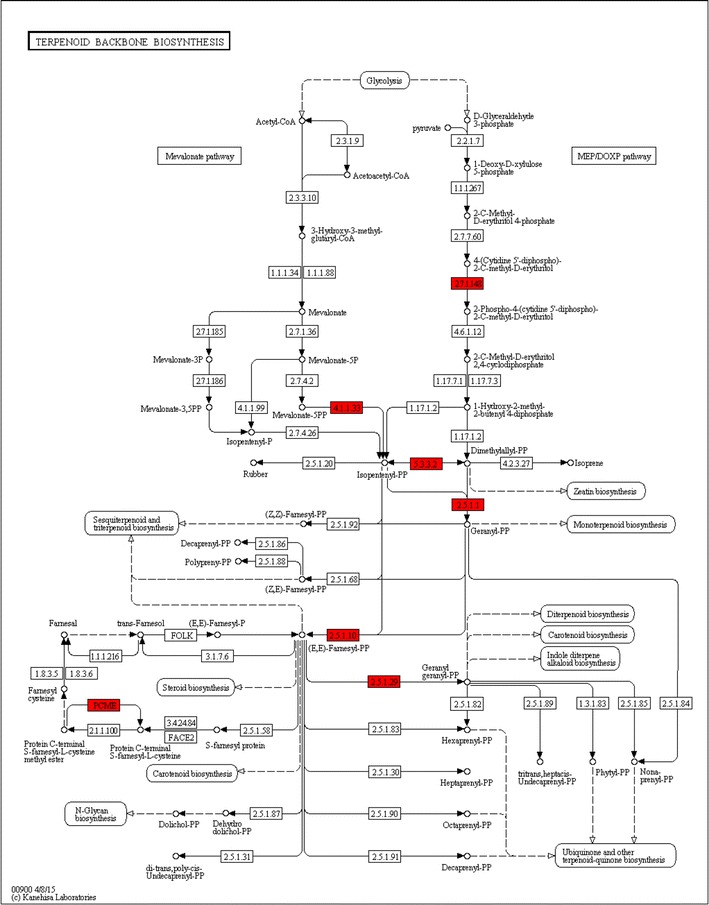
DEGs involved in terpenoid backbone biosynthesis

Using KEGG annotation, 517 new genes in *A. indica*, 21,238 *M. azedarach* genes and 763 DEGs were mapped to different KEGG pathways. The type classification of DEGs from the KEGG annotation results is shown in Fig. 2. Genes related to metabolism represented the largest proportion of DEGs, especially purine metabolism. Plant hormone signal transduction was the second largest category in the classification (Fig. 2). In relation to terpenoid synthesis, 135 unigenes participated in terpenoid backbone biosynthesis, 32 in sesquiterpenoid and triterpenoid biosynthesis, 29 in monoterpenoid biosynthesis and 50 in diterpenoid biosynthesis in *M. azedarach*, while 106, 52, 34 and 84 genes were involved in the corresponding biosynthetic processes in *A. indica*, respectively. Notably, only one new gene (new_gene 6030) was found to participate in terpenoid biosynthesis and all six DEGs involved in terpenoid backbone biosynthesis were up-regulated in *A. indica* (Fig. 3). It is likely that more metabolic flux is transfered into terpenoid synthesis in *A. indica*.

**Fig. 4:**
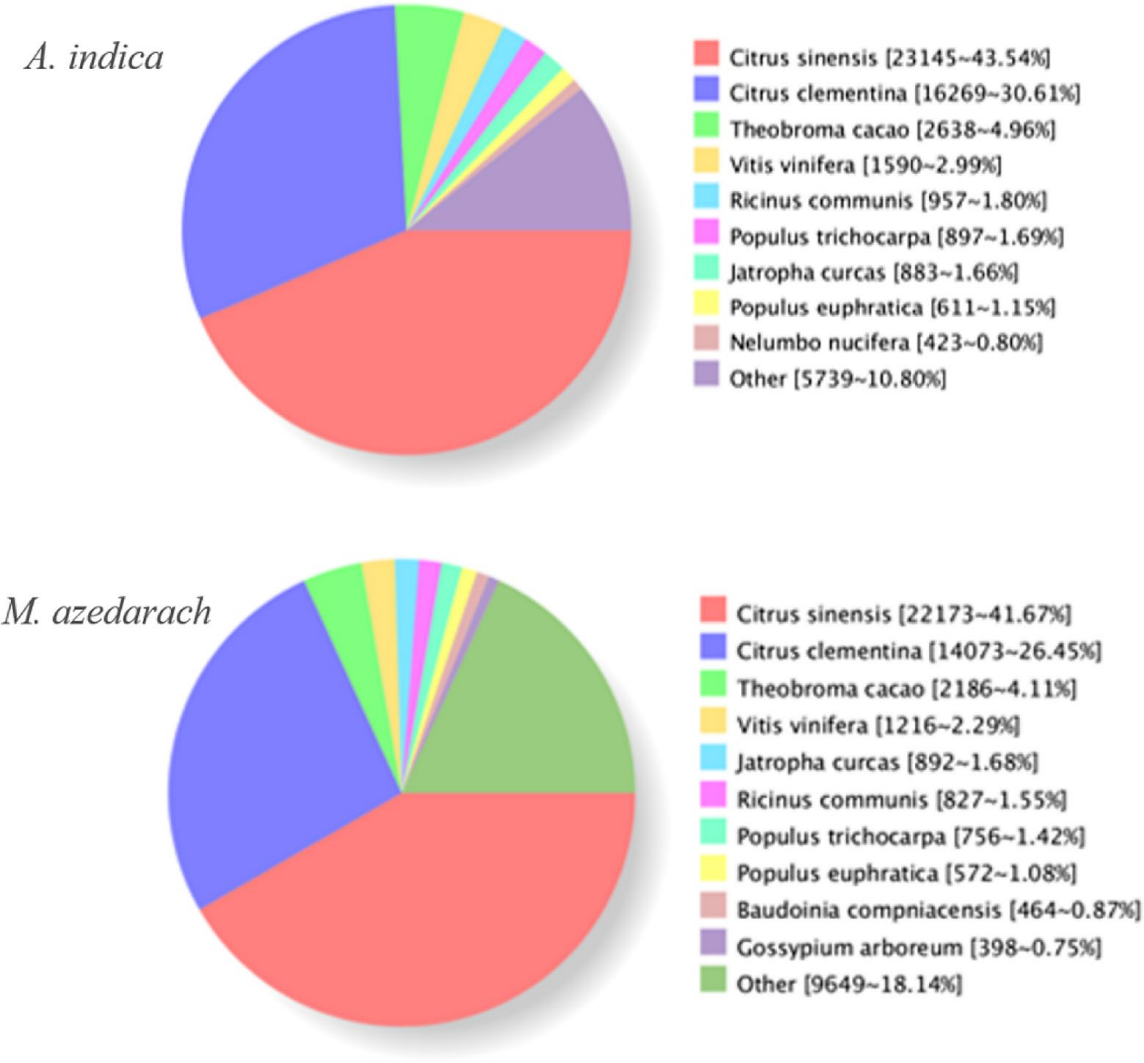
Homologous species distributions in *A. indica* and *M. azedarach*

The homologous species distribution from Nr annotation is shown in Fig. 4. The majority of genes of in *A. indica* and *M. azedarach* were most similar to homologous genes in *C. sinensis* (74.15 %) and *C. clementina* (68.12 %), which are *Rutaceae* plants. *Meliaceae* and *Rutaceae* both belong to *Rutineae* taxonomically, having a close evolutionary relationship.

### Chemical analysis of the azadirachtin and the total terpenoid content in *A. indica* and *M. azedarach* leaves

Using the standard curves (*y* = 5435.9*x* − 1501.2, *R*^2^ = 0.9996 for azadirachtin A; *y* = 3730.0*x* + 2696.2, *R*^2^ = 0.9995 for azadirachtin B), the total content of azadirachtin was calculated. Accordingly, 24.6, 24.05 and 51.77 mg/kg azadirachtin was detected from samples Y1–3, respectively, while azadirachtin was not detected in the K1–3 samples. Though azadirachtin was undetectable in *M. azedarach*, limonoids from *M. azedarach* also showed the activity to inhibit the development of flaviviruses and *Mycobacterium tubercolosis* (Sanna et al. 2015). Similarly, the total terpenoid content was calculated using the standard curve (y = 0.006x + 0.1064, *R*^2^ = 0.9775). The results showed that there was nearly 12.26 mg terpenoid in 0.5 g *A. indica* leaves, while 8.33 mg terpenoid in *M. azedarach* leaves.

## Conclusions

In this study, the transcriptome of *M. azedarach* was sequenced and analyzed for the first time, 225,972 transcripts and 91,607 unigenes were acquired, while 1179 new genes were detected from sequencing of *A. indica* libraries. Chemical analysis showed azadirachtin was only present in *A. indica* leaves; no azadirachtin or its derivatives were found in *M. azedarach*. The total terpenoid content assay showed there were 2.45 % terpenoid in *A. indica* leaves and 1.67 % terpenoid in *M. azedarach* leaves, respectively. These results will help us to research genes involved in the synthesis of bioactive compounds, and also associate the gene expression level with the metabolite content, especially terpenoid.

### Accession number

The Illumina HiSeq™ 2500 sequencing data from this study have been deposited in the NIH SRA database under the accession numbers: SRR3180937, SRR3181105 and SRR3181166 for *A. indica*, and SRR3183379, SRR3183380 and SRR3183381 for *M. azedarach*.

## Additional files

10.1186/s40064-016-2460-6 Saturation curve of the K libraries.
10.1186/s40064-016-2460-6 Orthologous genes with FPKM values.
10.1186/s40064-016-2460-6 Differentially expressed genes with annotation in the *A. indica* and *M. azedarach* libraries.
10.1186/s40064-016-2460-6 New genes with annotation in the *A. indica* libraries.
10.1186/s40064-016-2460-6 Unigenes with annotation in the *M. azedarach* libraries.
10.1186/s40064-016-2460-6 Functional categorization of new genes (a) and all genes (b) in *A. indica*, the unigenes in *M. azedarach* (c) and the DEGs (d) based on known genes in the GO database.

## Abbreviations

SRA: Short Read Archive database
NIH: National Institutes of Health
GO: Gene Ontology
KEGG: Kyoto Encyclopedia of Genes and Genomes
Nr: Non-redundant Protein databases
KOG: Eukaryotic Orthologous Groups
DEG: differentially expressed genes
FPKM: fragments per kilobase of transcript per million fragments mapped
RSEM: RNA-seq by expectation maximization
