# Supplementary figures and images for: Comparative analysis of the terpenoid biosynthesis pathway in Azadirachta indica and Melia azedarach by RNA-seq
Source: Springerplus. 2016 Jun 21;5(1):819. doi: 10.1186/s40064-016-2460-6 (PMC4916121; doi:10.1186/s40064-016-2460-6)

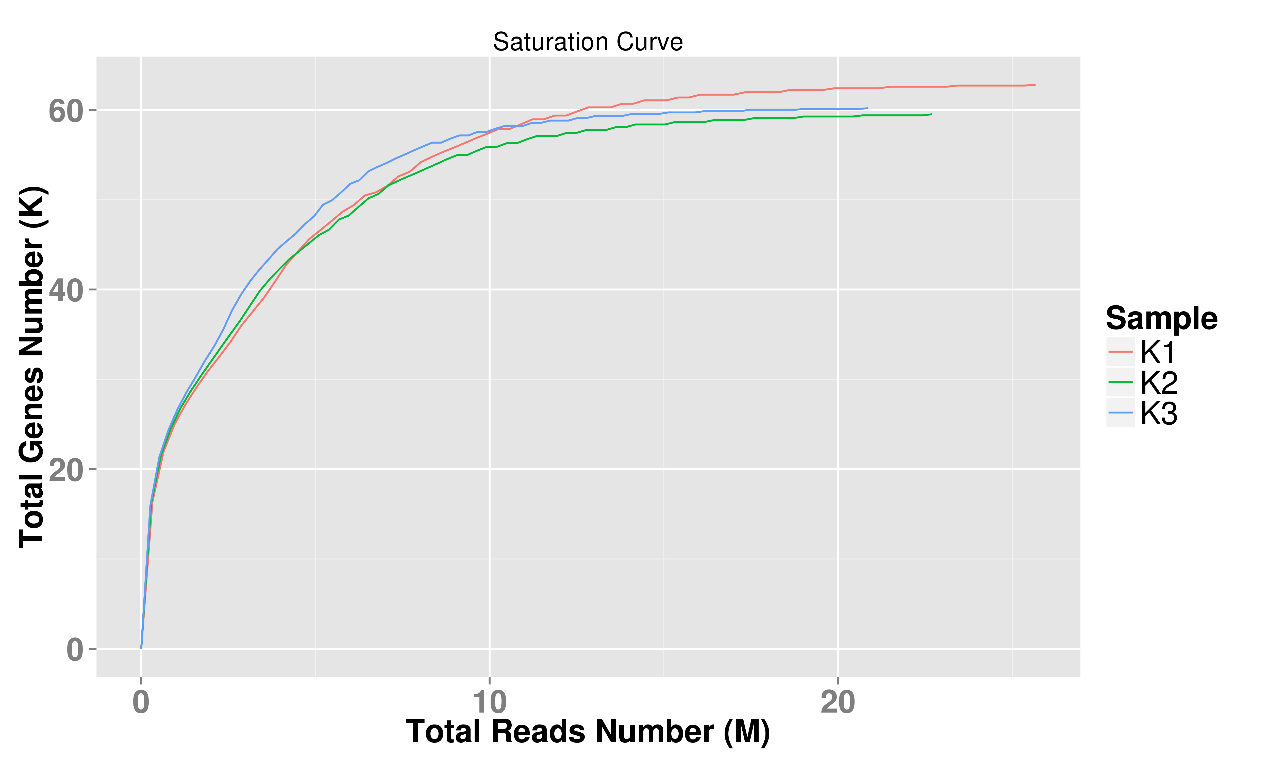


Figure S1 Saturation curve of K libraries

K1-3 stands for the three *M. azedarach* libraries.

Supplement: Supplementary file 1 — 10.1186/s40064-016-2460-6 Saturation curve of the K libraries. [file 40064_2016_2460_MOESM1_ESM.docx]
