# Supplementary material for: Comparative analysis of the terpenoid biosynthesis pathway in Azadirachta indica and Melia azedarach by RNA-seq
Source: Springerplus. 2016 Jun 21;5(1):819. doi: 10.1186/s40064-016-2460-6 (PMC4916121; doi:10.1186/s40064-016-2460-6)
Supplement: Supplementary file 6 — 10.1186/s40064-016-2460-6 Functional categorization of new genes (a) and all genes (b) in A. indica, the unigenes in M. azedarach (c) and the DEGs (d) based on known genes in the GO database. [file 40064_2016_2460_MOESM6_ESM.pdf]

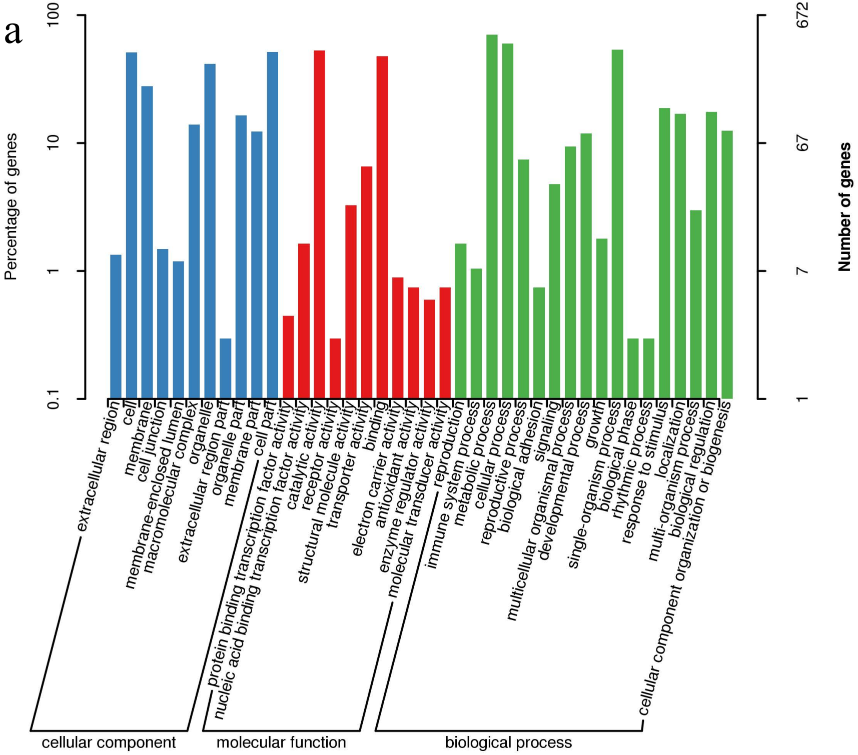

Figure S2a. Functional categorization of new genes in *A. indica* based on known genes in the GO database

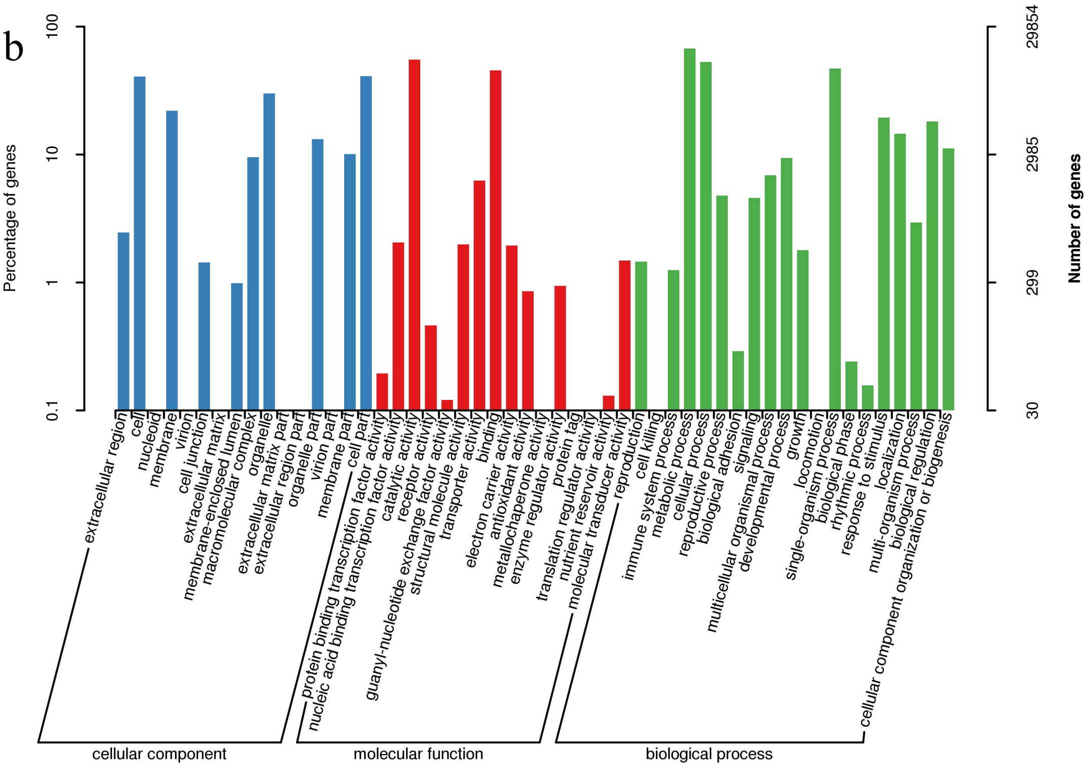

Figure S2b. Functional categorization of all genes in *A. indica* based on known genes in the GO database

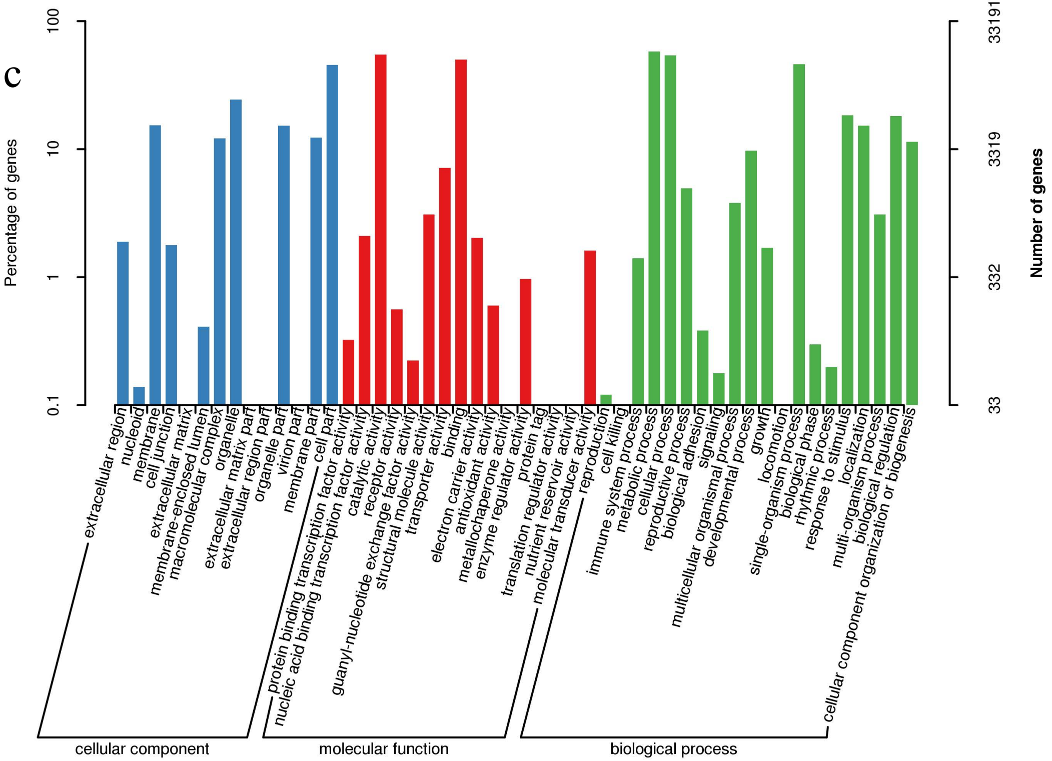

Figure S2c. Functional categorization of the unigenes in *M. azedarach* based on known genes in the GO database

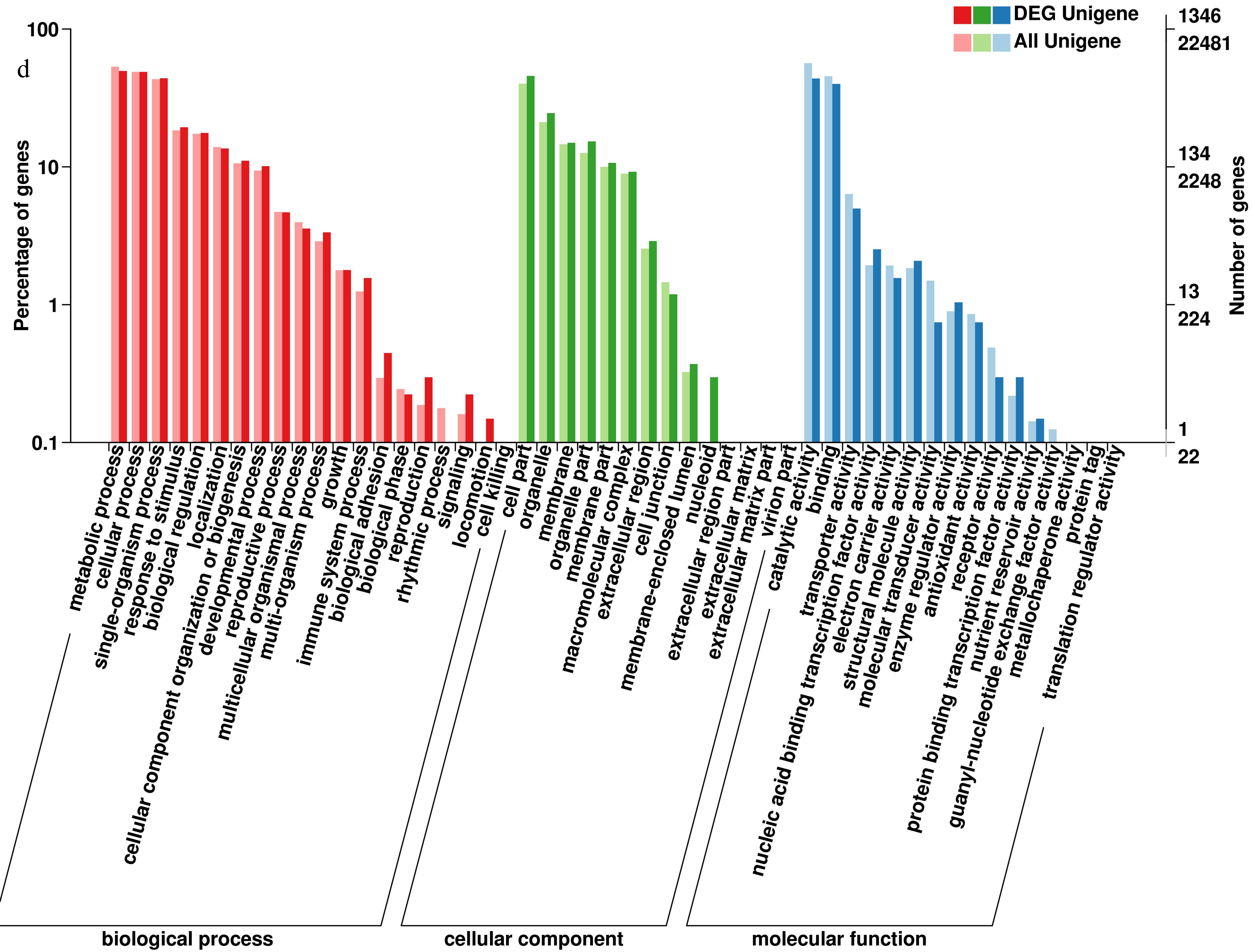

Figure S2d. Functional categorization of the DEGs in *M. azedarach* based on known genes in the GO database
